# Divergences Induced by the Cumulant and Partition Functions of Exponential Families and Their Deformations Induced by Comparative Convexity

**DOI:** 10.3390/e26030193

**Published:** 2024-02-23

**Authors:** Frank Nielsen

**Affiliations:** Sony Computer Science Laboratories, Tokyo 141-0022, Japan; frank.nielsen.x@gmail.com

**Keywords:** convex duality, exponential family, Bregman divergence, Jensen divergence, Bhattacharyya distance, Rényi divergence, α-divergences, comparative convexity, log convexity, exponential convexity, quasi-arithmetic means, information geometry

## Abstract

Exponential families are statistical models which are the workhorses in statistics, information theory, and machine learning, among others. An exponential family can either be normalized subtractively by its cumulant or free energy function, or equivalently normalized divisively by its partition function. Both the cumulant and partition functions are strictly convex and smooth functions inducing corresponding pairs of Bregman and Jensen divergences. It is well known that skewed Bhattacharyya distances between the probability densities of an exponential family amount to skewed Jensen divergences induced by the cumulant function between their corresponding natural parameters, and that in limit cases the sided Kullback–Leibler divergences amount to reverse-sided Bregman divergences. In this work, we first show that the α-divergences between non-normalized densities of an exponential family amount to scaled α-skewed Jensen divergences induced by the partition function. We then show how comparative convexity with respect to a pair of quasi-arithmetical means allows both convex functions and their arguments to be deformed, thereby defining dually flat spaces with corresponding divergences when ordinary convexity is preserved.

## 1. Introduction

In information geometry [1], any strictly convex and smooth function induces a dually flat space (DFS) with a canonical divergence which can be expressed in charts either as dual Bregman divergences [2] or equivalently as dual Fenchel–Young divergences [3]. For example, the cumulant function of an exponential family [4] (also called the free energy) generates a DFS, that is, an exponential family manifold [5] with the canonical divergence yielding the reverse Kullback–Leibler divergence. Another typical example of a strictly convex and smooth function generating a DFS is the negative entropy of a mixture family, that is, a mixture family manifold with the canonical divergence yielding the (forward) Kullback–Leibler divergence [3]. In addition, any strictly convex and smooth function induces a family of scaled skewed Jensen divergences [6,7], which in limit cases includes the sided forward and reverse Bregman divergences.

In Section 2, we present two equivalent approaches to normalizing an exponential family: first by its cumulant function, and second by its partition function. Because both the cumulant and partition functions are strictly convex and smooth, they induce corresponding families of scaled skewed Jensen divergences and Bregman divergences, with corresponding dually flat spaces and related statistical divergences.

In Section 3, we recall the well-known result that the statistical α-skewed Bhattacharyya distances between the *probability densities* of an exponential family amount to a scaled α-skewed Jensen divergence between their natural parameters. In Section 4, we prove that the α-divergences [8] between the *unnormalized densities* of a exponential family amount to scaled α-skewed Jensen divergence between their natural parameters (Proposition 5). More generally, we explain in Section 5 how to deform a convex function using comparative convexity [9]: When the ordinary convexity of the deformed convex function is preserved, we obtain new skewed Jensen divergences and Bregman divergences with corresponding dually flat spaces. Finally, Section 6 concludes this work with a discussion.

## 2. Dual Subtractive and Divisive Normalizations of Exponential Families

### 2.1. Natural Exponential Families

Let (X,A,μ) be a measure space [10], where X denotes the sample set (e.g., finite alphabet, N, $R^{d}$, space of positive-definite matrices ${\mathrm{Sym}}_{++}\left(d\right)$, etc.), A a σ-algebra on X (e.g., power set $2^{X}$, Borel σ-algebra B(X), etc.), and μ a positive measure (e.g., counting measure or Lebesgue measure) on the measurable space (X,A).

A natural exponential family [4,11] (commonly abbreviated as NEF [12]) is a set of probability distributions $P=\{P_{\theta }\;:\;\theta \in \Theta \}$ all dominated by μ such that their Radon–Nikodym densities $p_{\theta }\left(x\right)=\frac{dP_{\theta }}{d\mu }\left(x\right)$ can be expressed canonically as

(1) $$p_{\theta }\left(x\right)\propto {\tilde{p}}_{\theta }\left(x\right)=\exp\left(\sum_{i=1}^{m}\theta_{i}x_{i}\right),\;$$

where θ is called the natural parameter and $x=(x_{1},\ldots ,x_{m})$ denotes the linear sufficient statistic vector [11]. The order of the NEF [13] is *m*. When the parameter θ ranges in the full natural parameter space

$$\Theta =\left\{\theta \;:\;\int_{X}{\tilde{p}}_{\theta }\left(x\right)\;d\mu \left(x\right)<\infty \right\}\subset R^{m},\;$$

the family is called full. The NEF is said to be regular when Θ is topologically open.

The unnormalized positive density ${\tilde{p}}_{\theta }\left(x\right)$ is indicated with a tilde notation and the corresponding normalized probability density is obtained as $p_{\theta }\left(x\right)=\frac{1}{Z(\theta )}{\tilde{p}}_{\theta }\left(x\right)$, where $Z\left(\theta \right)=\int {\tilde{p}}_{\theta }\left(x\right)\;d\mu \left(x\right)$ is the Laplace transform of μ (the density normalizer). For example, the family of exponential distributions $E=\{\lambda e^{-\lambda x}\;:\;\lambda >0\}$ is an NEF with densities defined on the support $X=R_{\geq 0}=\left\{x\in R\;:\;x\geq 0\right\}$, natural parameter θ=−λ in $\Theta =R_{<0}=\left\{\theta \in R\;:\;\theta <0\right\}$, sufficient linear statistic *x*, and normalizer $Z\left(\theta \right)=-\frac{1}{\theta }$.

### 2.2. Exponential Families

More generally, exponential families include many well known distributions after reparameterization [4] of their ordinary parameter λ by θ(λ). The general canonical form of the densities of an exponential family is

(2) $$p_{\lambda }\left(x\right)\propto {\tilde{p}}_{\lambda }\left(x\right)=\exp\left(\langle \theta (\lambda ),t(x)\rangle \right)\;h\left(x\right),\;$$

where $t\left(x\right)=(t_{1}\left(x\right),\ldots ,t_{m}\left(x\right))$ are the sufficient statistic vector (such that $1,t_{1}\left(x\right),\ldots ,t_{m}\left(x\right)$ are linearly independent), h(x) is an auxiliary term used to define the base measure with respect to μ, and $\langle \cdot ,\cdot \rangle$ is an inner product (e.g., scalar product of $R^{m}$, trace product of symmetric matrices, etc.). By defining a new measure ν such that $\frac{d\mu }{d\nu }\left(x\right)=h\left(x\right)$, we may consider without loss of generality the densities ${\bar{p}}_{\lambda }\left(x\right)=\frac{dP_{\lambda }}{d\nu }\left(x\right)$ with h(x)=1.

For example, the Bernoulli distributions, Gaussian or normal distributions, Gamma and Beta distributions, Poisson distributions, Rayleigh distributions, and Weibull distributions with prescribed shape parameter are just a few examples of exponential families with the inner product on $R^{m}$ defined as the scalar product. The categorical distributions (i.e., discrete distributions on a finite alphabet sample space) form an exponential family as well [1]. Zero-centered Gaussian distributions and Wishart distributions are examples of exponential families parameterized by positive-definite matrices with inner products defined by the matrix trace product, which is $\langle A,B\rangle =\mathrm{tr}\left(AB\right)$.

Exponential families abound in statistics and machine learning. Any two probability measures *Q* and *R* with densities *q* and *r* with respect to a dominating measure, say, $\mu =\frac{Q+R}{2}$, define an exponential family

$$P_{Q,R}=\left\{p_{\lambda }\left(x\right)\propto q^{\lambda }\left(x\right)r^{1-\lambda }\left(x\right)\;:\;\lambda \in \left(0,1\right)\right\},\;$$

which is called the likelihood ratio exponential family [14], as the sufficient statistic is $t\left(x\right)=\log\frac{q(x)}{r(x)}$ (with auxiliary carrier term h(x)=r(x)), or the Bhattacharyya arc, as the cumulant function of $P_{Q,R}$ is expressed as the negative of the skewed Bhattacharyya distances [7,15].

In machine learning, undirected graphical models [16] and energy-based models [17], including Markov random fields [18] and conditional random fields, are exponential families [19]. Exponential families are universal approximators of smooth densities [20].

From a theoretical standpoint, it is often enough to consider (without loss of generality) natural exponential families with densities expressed as in Equation (1). However, here we consider generic exponential families with the densities expressed in Equation (2) in order to report common examples encountered in practice, such as the multivariate Gaussian family [21].

When the natural parameter space Θ is not full but rather parameterized by $\lambda =c(\lambda^{\prime })$ for $\lambda^{\prime }\in \Lambda^{\prime }$ with $\dim(\Lambda^{\prime })<m$ and a smooth function c(u), the exponential family is called a curved exponential family [1]. For example, the special family of normal distributions $\left\{p_{\mu ,\sigma^{2}=\mu^{2}}\;:\;\mu \in R\right\}$ is a curved exponential family with u=μ and $c\left(u\right)=\left(u,u^{2}\right)$ [1].

### 2.3. Normalizations of Exponential Families

Recall that ${\tilde{p}}_{\theta }\left(x\right)=\exp\left(\langle \theta ,t(x)\rangle \right)\;h\left(x\right)$ denotes the unnormalized density expressed using the natural parameter θ=θ(λ). We can normalize ${\tilde{p}}_{\theta }\left(x\right)$ using either the partition function Z(θ) or equivalently using the cumulant function F(θ), as follows:

(3) $$\begin{array}{ccc} p_{\theta }\left(x\right) & = & \frac{\exp(\langle \theta ,t(x)\rangle )}{Z(\theta )}\;h\left(x\right), \end{array}$$

(4) $$\begin{array}{ccc} & = & \exp\left(\langle \theta ,t(x)\rangle -F\left(\theta \right)+k\left(x\right)\right),\; \end{array}$$

where h(x)=exp(k(x)), $Z\left(\theta \right)=\int {\tilde{p}}_{\theta }\left(x\right)d\mu \left(x\right)$, and $F\left(\theta \right)=\log Z\left(\theta \right)=\log\int {\tilde{p}}_{\theta }\left(x\right)d\mu \left(x\right)$. Thus, the logarithm and exponential functions allow conversion to and from the dual normalizers *Z* and *F*:

Z(θ)=exp(F(θ))⇔F(θ)=logZ(θ).

We may view Equation (3) as an exponential tilting [13] of density h(x)dμ(x).

In the context of λ-deformed exponential families [22] which generalize exponential families, the function Z(θ) is called the divisive normalization factor (Equation (3)) and the function F(θ) is called the subtractive normalization factor (Equation (4)). Notice that F(θ) is called the cumulant function because when $X∼p_{\theta }\left(x\right)$ is a random variable following a probability distribution of an exponential family, the function F(θ) appears in the cumulant generating function of *X*: $K_{X}\left(t\right)=\log E_{X}\left[e^{\langle t,X\rangle }\right]=F\left(\theta +t\right)-F\left(\theta \right)$. In statistical physics, the cumulant function is called the log-normalizer or log-partition function. Because Z>0 and F=logZ, we can deduce that F≥Z, as logx≤x for x>0.

It is well known that the cumulant function F(θ) is a strictly convex function and that the partition function Z(θ) is strictly log-convex [11].

**Proposition** **1** ([11]). *The natural parameter space Θ of an exponential family is convex.*

**Proposition** **2** ([11]). *The cumulant function F(θ) is strictly convex and the partition function Z(θ) is positive and strictly log-convex.*

It can be shown that the cumulant and partition functions are smooth $C^{\infty }$ analytic functions [4]. A remarkable property is that strictly log-convex functions are also strictly convex.

**Proposition** **3** ([23], Section 3.5). *A strictly log-convex function $Z:\Theta \subset R^{m}\to R$ is strictly convex.*

**Figure 1:**
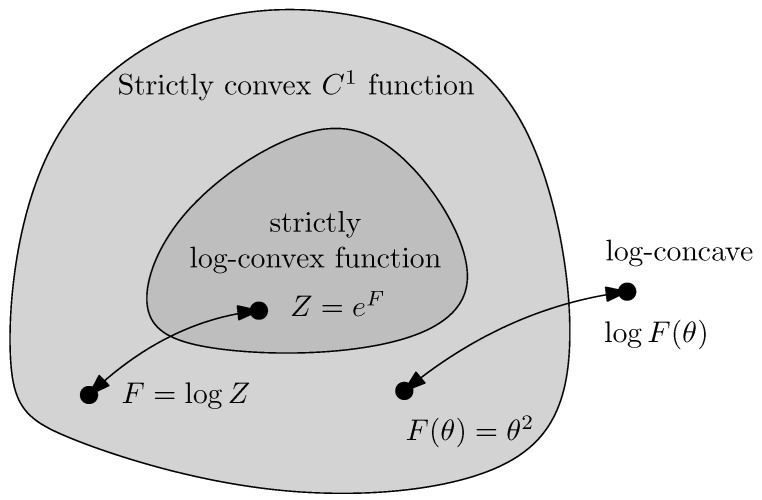
Strictly log-convex functions form a proper subset of strictly convex functions.

The converse of Proposition 3 is not necessarily true, however; certain convex functions are not log-convex, and as such the class of strictly log-convex functions is a proper subclass of strictly convex functions. For example, $\theta^{2}$ is convex but log-concave, as ${\left(\log\theta^{2}\right)}^{\prime \prime }=-\frac{2}{\theta^{2}}<0$ (Figure 1).

**Remark** **1.** *Because Z=exp(F) is strictly convex (Proposition* 3*), F is exponentially convex.*

**Definition** **1.** *The cumulant function F and partition function Z of a regular exponential family are both strictly convex and smooth functions inducing a pair of dually flat spaces with corresponding Bregman divergences* [2] *$B_{F}$ (i.e., $B_{\log Z}$) and $B_{Z}$ (i.e., $B_{\exp F}$):*

(5) $$\begin{array}{ccc} B_{Z}\left(\theta_{1}:\theta_{2}\right) & = & Z\left(\theta_{1}\right)-Z\left(\theta_{2}\right)-\langle \theta_{1}-\theta_{2},\nabla Z\left(\theta_{2}\right)\rangle \geq 0, \end{array}$$

(6) $$\begin{array}{ccc} B_{\log Z}\left(\theta_{1}:\theta_{2}\right) & = & \log\left(\frac{Z(\theta_{1})}{Z(\theta_{2})}\right)-\langle \theta_{1}-\theta_{2},\frac{\nabla Z(\theta_{2})}{Z(\theta_{2})}\rangle \geq 0, \end{array}$$

*along with a pair of families of skewed Jensen divergences $J_{F,\alpha }$ and $J_{Z,\alpha }$:*

(7) $$\begin{array}{ccc} J_{Z,\alpha }\left(\theta_{1}:\theta_{2}\right) & = & \alpha Z\left(\theta_{1}\right)+\left(1-\alpha \right)Z\left(\theta_{2}\right)-Z\left(\alpha \theta_{1}+\left(1-\alpha \right)\theta_{2}\right)\geq 0, \end{array}$$

(8) $$\begin{array}{ccc} J_{\log Z,\alpha }\left(\theta_{1}:\theta_{2}\right) & = & \log\frac{Z{\left(\theta_{1}\right)}^{\alpha }Z{\left(\theta_{2}\right)}^{1-\alpha }}{Z(\alpha \theta_{1}+\left(1-\alpha \right)\theta_{2})}\geq 0. \end{array}$$

For a strictly convex function F(θ), we define the symmetric Jensen divergence as follows:

$$J_{F}\left(\theta_{1},\theta_{2}\right)=J_{F,\frac{1}{2}}\left(\theta_{1}:\theta_{2}\right)=\frac{F\left(\theta_{1}\right)+F\left(\theta_{2}\right)}{2}-F\left(\frac{\theta_{1}+\theta_{2}}{2}\right).$$

Let $B_{\Theta }$ denote the set of real-valued strictly convex and differentiable functions defined on an open set Θ, called Bregman generators. We may equivalently consider the set of strictly concave and differentiable functions G(θ) and let F(θ)=−G(θ); see [24] (Equation (1)).

**Remark** **2.** *The non-negativeness of the Bregman divergences for the cumulant and partition functions define the criteria for checking the strict convexity or log-convexity of a $C^{1}$ function:*

$$\begin{array}{ccc} F(\theta )\;is\;strictly\;convex & \Leftrightarrow & \forall \theta_{1}\neq \theta_{2},B_{F}\left(\theta_{1}:\theta_{2}\right)>0,\\ & \Leftrightarrow & \forall \theta_{1}\neq \theta_{2},F\left(\theta_{1}\right)>F\left(\theta_{2}\right)+\langle \theta_{1}-\theta_{2},\nabla F\left(\theta \right)\rangle , \end{array}$$

*and*

$$\begin{array}{ccc} Z(\theta )\;is\;strictly\;log-convex & \Leftrightarrow & \forall \theta_{1}\neq \theta_{2},B_{\log Z}\left(\theta_{1}:\theta_{2}\right)>0,\\ & \Leftrightarrow & \forall \theta_{1}\neq \theta_{2},\log Z\left(\theta_{1}\right)>\log Z\left(\theta_{2}\right)+\langle \theta_{1}-\theta_{2},\frac{\nabla Z(\theta_{2})}{Z(\theta_{2})}\rangle . \end{array}$$

The forward Bregman divergence $B_{F}\left(\theta_{1}:\theta_{2}\right)$ and reverse Bregman divergence $B_{F}\left(\theta_{2}:\theta_{1}\right)$ can be unified with the α-skewed Jensen divergences by rescaling $J_{F,\alpha }$ and allowing α to range in R [6,7]:

(9) $$J_{F,\alpha }^{s}\left(\theta_{1}:\theta_{2}\right)=\left\{\begin{array}{cc} \frac{1}{\alpha (1-\alpha )}J_{F,\alpha }\left(\theta_{1}:\theta_{2}\right), & \alpha \in R\setminus \{0,1\},\\ B_{F}\left(\theta_{1}:\theta_{2}\right), & \alpha =0,\\ 4\;J_{F}\left(\theta_{1},\theta_{2}\right), & \alpha =\frac{1}{2},\\ B_{F}^{*}\left(\theta_{1}:\theta_{2}\right)=B_{F}\left(\theta_{2}:\theta_{1}\right), & \alpha =1. \end{array}\right.,$$

where ${B_{F}}^{*}$ denotes the reverse Bregman divergence obtained by swapping the parameter order (reference duality [6]): ${B_{F}}^{*}\left(\theta_{1}:\theta_{2}\right)=B_{F}\left(\theta_{2}:\theta_{1}\right)$.

**Remark** **3.** *Alternatively, we may rescale $J_{F}$ by a factor $\kappa \left(\alpha \right)=\frac{1}{\alpha \left(1-\alpha \right)4^{4\alpha (1-\alpha )}}$, i.e., $J_{F,\alpha }^{\bar{s}}\left(\theta_{1}:\theta_{2}\right)=\kappa \left(\alpha \right)\;J_{F,\alpha }\left(\theta_{1}:\theta_{2}\right)$ such that $\kappa (\frac{1}{2})=1$ and $J_{F,\frac{1}{2}}^{\bar{s}}\left(\theta_{1}:\theta_{2}\right)=J_{F}\left(\theta_{1},\theta_{2}\right)$.*

Next, in Section 3 we first recall the connections between these Jensen and Bregman divergences, which are divergences between parameters, and the statistical divergence counterparts between probability densities. Then, in Section 4 we introduce the novel connections between these parameter divergences and α-divergences between unnormalized densities.

## 3. Divergences Related to the Cumulant Function

Consider the scaled α-skewed Bhattacharyya distances [7,15] between two probability densities p(x) and q(x):

$$D_{B,\alpha }^{s}\left(p:q\right)=-\frac{1}{\alpha (1-\alpha )}\log\int p^{\alpha }q^{1-\alpha }d\mu ,\;\alpha \in R\setminus \left\{0,1\right\}.\;$$

The scaled α-skewed Bhattacharyya distances can additionally be interpreted as Rényi divergences [25] scaled by $\frac{1}{\alpha }$: $D_{B,\alpha }^{s}\left(p:q\right)=\frac{1}{\alpha }D_{R,\alpha }\left(p:q\right)$, where the Rényi α-divergences are defined by

$$D_{R,\alpha }\left(p:q\right)=\frac{1}{\alpha -1}\log\int p^{\alpha }q^{1-\alpha }\;d\mu .$$

The Bhattacharyya distance $D_{B}\left(p,q\right)=-\log\int \sqrt{pq}d\mu$ corresponds to one-fourth of $D_{B,\frac{1}{2}}^{s}\left(p:q\right)$: $D_{B}\left(p,q\right)=\frac{1}{4}\;D_{B,\frac{1}{2}}^{s}\left(p:q\right)$. Because $D_{B,\alpha }^{s}$ tends to the Kullback–Leibler divergence $D_{\mathrm{KL}}$ when α→1 and to the reverse Kullback–Leibler divergence ${D_{\mathrm{KL}}}^{*}$ when α→0, we have

$$D_{B,\alpha }^{s}\left(p:q\right)=\left\{\begin{array}{cc} -\frac{1}{\alpha (1-\alpha )}\;\log\int p^{\alpha }q^{1-\alpha }d\mu , & \alpha \in R\setminus \{0,1\},\\ D_{\mathrm{KL}}\left(p:q\right), & \alpha =1,\\ 4\;D_{B}\left(p,q\right) & \alpha =\frac{1}{2},\\ {D_{\mathrm{KL}}}^{*}\left(p:q\right)=D_{\mathrm{KL}}\left(q:p\right) & \alpha =0. \end{array}\right.$$

When both probability densities belong to the same exponential family $E=\{p_{\theta }\left(x\right)\;:\;\theta \in \Theta \}$ with cumulant F(θ), we have the following proposition.

**Proposition** **4** ([7]). *The scaled α-skewed Bhattacharyya distances between two probability densities $p_{\theta_{1}}$ and $p_{\theta_{2}}$ of an exponential family amount to the scaled α-skewed Jensen divergence between their natural parameters:*

(10) $$D_{B,\alpha }^{s}\left(p_{\theta_{1}}:p_{\theta_{2}}\right)=J_{F,\alpha }^{s}\left(\theta_{1},\theta_{2}\right).$$

**Proof.**  The proof follows by first considering the α-skewed Bhattacharyya similarity coefficient $\rho_{\alpha }\left(p,q\right)=\int p^{\alpha }q^{1-\alpha }d\mu$.

$$\begin{array}{ccc} \rho_{\alpha }\left(p_{\theta_{1}}:p_{\theta_{2}}\right) & = & \int \exp{\left(\langle \theta_{1},x\rangle -F\left(\theta_{1}\right)\right)}^{\alpha }\;\exp{\left(\langle \theta_{2},x\rangle -F\left(\theta_{2}\right)\right)}^{1-\alpha }d\mu ,\\ & = & \int \exp\left(\langle \alpha \theta_{1}+\left(1-\alpha \right)\theta_{2}),x\rangle \right)\;\exp\left(-(\alpha F\left(\theta_{1}\right)+\left(1-\alpha \right)F\left(\theta_{2}\right))\right)d\mu . \end{array}$$

Multiplying the last equation by $\exp\left(F\left(\alpha \theta_{1}+\left(1-\alpha \right)\theta_{2}\right)\right)\;\exp\left(-F\left(\alpha \theta_{1}+\left(1-\alpha \right)\theta_{2}\right)\right)=\exp\left(0\right)=1$ with $\bar{\theta }=\alpha \theta_{1}+\left(1-\alpha \right)\theta_{2}$, we obtain

$$\rho_{\alpha }\left(p_{\theta_{1}}:p_{\theta_{2}}\right)=\exp(-\left(\alpha F\left(\theta_{1}\right)+\left(1-\alpha \right)F\left(\theta_{2}\right)\right)\exp\left(F\left(\bar{\theta }\right)\right)\int \exp\left(\langle \bar{\theta },x\rangle -F\left(\bar{\theta }\right)\right)d\mu .$$

Because $\bar{\theta }\in \Theta$, we have $\int \exp(\langle \bar{\theta },x\rangle -F\left(\bar{\theta }\right))d\mu =1$; therefore, we obtain

$$\rho_{\alpha }\left(p_{\theta_{1}}:p_{\theta_{2}}\right)=\exp\left(-J_{F,\alpha }\left(\theta_{1}:\theta_{2}\right)\right).$$

□

For practitioners in machine learning, it is well known that the Kullback–Leibler divergence between two probability densities $p_{\theta_{1}}$ and $p_{\theta_{2}}$ of an exponential family amounts to a Bregman divergence for the cumulant generator on a swapped parameter order (e.g., [26,27]):

$$D_{\mathrm{KL}}\left(p_{\theta_{1}}:p_{\theta_{2}}\right)=B_{F}\left(\theta_{2}:\theta_{1}\right).\;$$

This is a particular instance of Equation (10) obtained for α=1:

$$D_{B,1}^{s}\left(p_{\theta_{1}}:p_{\theta_{2}}\right)=J_{F,1}^{s}\left(\theta_{1},\theta_{2}\right).\;$$

This formula has been further generalized in [28] by considering truncations of exponential family densities. Let $X_{1}\subseteq X_{2}\subseteq X$ and $E_{1}=\left\{1_{X_{1}}\left(x\right)\;p_{\theta }\left(x\right)\right\}$, $E_{2}=\left\{1_{X_{2}}\left(x\right)\;q_{\theta^{\prime }}\left(x\right)\right\}$ be two truncated families of X with corresponding cumulant functions

$$F_{1}\left(\theta \right)=\log\int_{X_{1}}\exp\left(\langle t(x),\theta \rangle \right)d\mu$$

and

$$F_{2}\left(\theta^{\prime }\right)=\log\int_{X_{2}}\exp\left(\langle t(x),\theta \rangle \right)d\mu \geq F_{1}\left(\theta^{\prime }\right).$$

Then, we have

$$\begin{array}{ccc} D_{\mathrm{KL}}\left(p_{\theta_{1}}:q_{\theta_{2}^{\prime }}\right) & = & B_{F_{2},F_{1}}\left(\theta_{2}^{\prime }:\theta_{1}\right),\\ & = & F_{2}\left(\theta_{2}^{\prime }\right)-F_{1}\left(\theta_{1}\right)-\langle \theta_{2}^{\prime }-\theta_{1},\nabla F_{1}\left(\theta_{1}\right)\rangle . \end{array}$$

Truncated exponential families are normalized exponential families which may not be regular [29], i.e., the parameter space Θ may not be open.

## 4. Divergences Related to the Partition Function

Certain exponential families have intractable cumulant/partition functions (e.g., exponential families with sufficient statistics $t\left(x\right)=\left(x,x^{2},\ldots ,x^{m}\right)$ for high degrees *m* [20]) or cumulant/partition functions which require exponential time to compute [30] (e.g., graphical models [16], high-dimensional grid sample spaces, energy-based models [17] in deep learning, etc.). In such cases, the maximum likelihood estimator (MLE) cannot be used to infer the natural parameter of exponential densities. Many alternative methods have been proposed to handle such exponential families with untractable partition functions, e.g., score matching [31] or divergence-based inference [32,33]). Thus, it is important to consider dissimilarities between non-normalized statistical models.

The squared Hellinger distance [1] between two positive potentially unnormalized densities $\tilde{p}$ and $\tilde{q}$ is defined by

$$\begin{array}{ccc} D_{H}^{2}\left(\tilde{p},\tilde{q}\right) & = & \frac{1}{2}\int {\left(\sqrt{\tilde{p}}-\sqrt{\tilde{q}}\right)}^{2}\;d\mu ,\\ & = & \frac{\int \tilde{p}d\mu +\int \tilde{q}d\mu }{2}-\int \sqrt{\tilde{p}\tilde{q}}d\mu .\; \end{array}$$

Notice that the Hellinger divergence can be interpreted as the integral of the difference between the arithmetical mean $A\left(\tilde{p},\tilde{q}\right)=\frac{\tilde{p}+\tilde{q}}{2}$ minus the geometrical mean $G\left(\tilde{p},\tilde{q}\right)=\sqrt{\tilde{p}\tilde{q}}$ of the densities: $D_{H}^{2}\left(\tilde{p},\tilde{q}\right)=\int \left(A\left(\tilde{p},\tilde{q}\right)-G\left(\tilde{p},\tilde{q}\right)\right)d\mu$. This further proves that $D_{H}\left(\tilde{p},\tilde{q}\right)\geq 0$, as A≥G. The Hellinger distance $D_{H}$ satisfies the metric axioms of distances.

When considering unnormalized densities ${\tilde{p}}_{\theta_{1}}=\exp\left(\langle t\left(x\right),\theta_{1}\rangle \right)$ and ${\tilde{p}}_{\theta_{2}}=\exp\left(\langle t\left(x\right),\theta_{2}\rangle \right)$ of an exponential family E with a partition function $Z\left(\theta \right)=\int {\tilde{p}}_{\theta }\;d\mu$, we obtain

(11) $$D_{H}^{2}\left({\tilde{p}}_{\theta_{1}},{\tilde{p}}_{\theta_{2}}\right)=\frac{Z\left(\theta_{1}\right)+Z\left(\theta_{2}\right)}{2}-Z\left(\frac{\theta_{1}+\theta_{2}}{2}\right)=J_{Z}\left(\theta_{1},\theta_{2}\right),$$

as $\sqrt{{\tilde{p}}_{\theta_{1}}{\tilde{p}}_{\theta_{2}}}={\tilde{p}}_{\frac{\theta_{1}+\theta_{2}}{2}}$.

The Kullback–Leibler divergence [1] as extended to two positive densities $\tilde{p}$ and $\tilde{q}$ is defined by

(12) $$D_{\mathrm{KL}}\left(\tilde{p}:\tilde{q}\right)=\int \left(\tilde{p}\log\frac{\tilde{p}}{\tilde{q}}+\tilde{q}-\tilde{p}\right)d\mu .$$

When considering unnormalized densities ${\tilde{p}}_{\theta_{1}}$ and ${\tilde{p}}_{\theta_{2}}$ of E, we obtain

(13) $$\begin{array}{ccc} D_{\mathrm{KL}}\left({\tilde{p}}_{\theta_{1}}:{\tilde{p}}_{\theta_{2}}\right) & = & \int \left({\tilde{p}}_{\theta_{1}}\left(x\right)\log\frac{{\tilde{p}}_{\theta_{1}}\left(x\right)}{{\tilde{p}}_{\theta_{2}}\left(x\right)}+{\tilde{p}}_{\theta_{2}}\left(x\right)-{\tilde{p}}_{\theta_{1}}\left(x\right)\right)d\mu \left(x\right), \end{array}$$

(14) $$\begin{array}{ccc} & = & \int \left(e^{\langle t\left(x\right),\theta_{1}\rangle }\langle \theta_{1}-\theta_{2},t\left(x\right)\rangle +e^{\langle t\left(x\right),\theta_{2}\rangle }-e^{\langle t\left(x\right),\theta_{1}\rangle }\right)\;d\mu \left(x\right), \end{array}$$

(15) $$\begin{array}{ccc} & = & \langle \int t\left(x\right)e^{\langle t\left(x\right),\theta_{1}\rangle }d\mu \left(x\right),\theta_{1}-\theta_{2}\rangle +Z\left(\theta_{2}\right)-Z\left(\theta_{1}\right), \end{array}$$

(16) $$\begin{array}{ccc} & = & \langle \theta_{1}-\theta_{2},\nabla Z\left(\theta_{1}\right)\rangle +Z\left(\theta_{2}\right)-Z\left(\theta_{1}\right)=B_{Z}\left(\theta_{2}:\theta_{1}\right), \end{array}$$

as $\nabla Z\left(\theta \right)=\int t\left(x\right){\tilde{p}}_{\theta }\left(x\right)d\mu \left(x\right)$. Let $D_{\mathrm{KL}}^{*}\left(\tilde{p}:\tilde{q}\right)=D_{\mathrm{KL}}\left(\tilde{q}:\tilde{p}\right)$ denote the reverse KLD.

More generally, the family of α-divergences [1] between the unnormalized densities $\tilde{p}$ and $\tilde{q}$ is defined for α∈R by

$$D_{\alpha }\left(\tilde{p}:\tilde{q}\right)=\left\{\begin{array}{cc} \frac{1}{\alpha (1-\alpha )}\int \left(\alpha \tilde{p}+\left(1-\alpha \right)\tilde{q}-{\tilde{p}}^{\alpha }{\tilde{q}}^{1-\alpha }\right)d\mu , & \alpha \notin \{0,1\}\\ D_{\mathrm{KL}}^{*}\left(\tilde{p}:\tilde{q}\right)=D_{\mathrm{KL}}\left(\tilde{q}:\tilde{p}\right) & \alpha =0,\\ 4\;D_{H}^{2}\left(\tilde{p},\tilde{q}\right) & \alpha =\frac{1}{2},\\ D_{\mathrm{KL}}\left(\tilde{p}:\tilde{q}\right) & \alpha =1. \end{array}\right.$$

We now have $D_{\alpha }^{*}\left(\tilde{p}:\tilde{q}\right)=D_{\alpha }\left(\tilde{q}:\tilde{p}\right)=D_{1-\alpha }\left(\tilde{p}:\tilde{q}\right)$, and the α-divergences are homogeneous divergences of degree 1. For all λ>0, we have $D_{\alpha }\left(\lambda \tilde{q}:\lambda \tilde{p}\right)=\lambda \;D_{\alpha }\left(\tilde{q}:\tilde{p}\right)$. Moreoever, because $\alpha \tilde{p}+\left(1-\alpha \right)\tilde{q}-{\tilde{p}}^{\alpha }{\tilde{q}}^{1-\alpha }$ can be expressed as the difference of the weighted arithmetic mean minus the weighted geometric mean $A\left(\tilde{p},\tilde{q};\alpha ;1-\alpha \right)-G\left(\tilde{p},\tilde{q};\alpha ;1-\alpha \right)$, it follows from the arithmetical–geometrical mean inequality that we have $D_{\alpha }\left(\tilde{p}:\tilde{q}\right)\geq 0$.

When considering unnormalized densities ${\tilde{p}}_{\theta_{1}}$ and ${\tilde{p}}_{\theta_{2}}$ of E, we obtain

$$D_{\alpha }\left({\tilde{p}}_{\theta_{1}}:{\tilde{p}}_{\theta_{2}}\right)=\left\{\begin{array}{cc} \frac{1}{\alpha (1-\alpha )}J_{Z,\alpha }\left(\theta_{1}:\theta_{2}\right), & \alpha \notin \{0,1\}\\ B_{Z}\left(\theta_{1}:\theta_{2}\right) & \alpha =0,\\ 4\;J_{Z}\left(\theta_{1},\theta_{2}\right) & \alpha =\frac{1}{2},\\ B_{Z}^{*}\left(\theta_{1}:\theta_{2}\right)=B_{Z}\left(\theta_{2}:\theta_{1}\right) & \alpha =1 \end{array}.\right.$$

**Proposition** **5.** *The α-divergences between the unnormalized densities of an exponential family amount to scaled α-Jensen divergences between their natural parameters for the partition function*

$$D_{\alpha }\left({\tilde{p}}_{\theta_{1}}:{\tilde{p}}_{\theta_{2}}\right)=J_{Z,\alpha }^{s}\left(\theta_{1}:\theta_{2}\right).$$

*When α∈{0,1}, the oriented Kullback–Leibler divergences between unnormalized exponential family densities amount to reverse Bregman divergences on their corresponding natural parameters for the partition function*

$$D_{\mathrm{KL}}\left(\tilde{p}:\tilde{q}\right)=B_{Z}\left(\theta_{2}:\theta_{1}\right).$$

**Proof.**  For α∉{0,1}, consider

$$D_{\alpha }\left({\tilde{p}}_{\theta_{1}}:{\tilde{p}}_{\theta_{2}}\right)=\frac{1}{\alpha (1-\alpha )}\int \left(\alpha {\tilde{p}}_{\theta_{1}}+\left(1-\alpha \right){\tilde{p}}_{\theta_{2}}-{\tilde{p}}_{\theta_{1}}^{\alpha }{\tilde{p}}_{\theta_{2}}^{1-\alpha }\right)d\mu .$$

Here, we have $\int \alpha {\tilde{p}}_{\theta_{1}}\;d\mu =\alpha Z\left(\theta_{1}\right)$, $\int \left(1-\alpha \right){\tilde{p}}_{\theta_{2}}\;d\mu =\left(1-\alpha \right)Z\left(\theta_{2}\right)$ and $\int {\tilde{p}}_{\theta_{1}}^{\alpha }{\tilde{p}}_{\theta_{2}}^{1-\alpha }d\mu =\int {\tilde{p}}_{\alpha \theta_{1}+\left(1-\alpha \right)\theta_{2}}d\mu =Z\left(\alpha \theta_{1}+\left(1-\alpha \right)\theta_{2}\right)$. It follows that

$$D_{\alpha }\left({\tilde{p}}_{\theta_{1}}:{\tilde{p}}_{\theta_{2}}\right)=\frac{1}{\alpha (1-\alpha )}J_{Z,\alpha }\left(\theta_{1}:\theta_{2}\right)=J_{Z,\alpha }^{s}\left(\theta_{1}:\theta_{2}\right).$$

□

Notice that the KLD extended to unnormalized densities can be written as a generalized relative entropy, i.e., it can be obtained as the difference of the extended cross-entropy minus the extended entropy (self cross-entropy):

$$\begin{array}{ccc} D_{\mathrm{KL}}\left(\tilde{p}:\tilde{q}\right) & = & H^{\times }\left(\tilde{p}:\tilde{q}\right)-H\left(\tilde{p}\right),\\ & = & \int \left(\tilde{p}\log\frac{\tilde{p}}{\tilde{q}}+\tilde{q}-\tilde{p}\right)d\mu \end{array}$$

with

$$H^{\times }\left(\tilde{p}:\tilde{q}\right)=\int \left(\tilde{p}\left(x\right)\log\frac{1}{\tilde{q}\left(x\right)}+\tilde{q}\left(x\right)\right)d\mu \left(x\right)-1$$

and

$$H\left(\tilde{p}\right)=H^{\times }\left(\tilde{p}:\tilde{p}\right)=\int \left(\tilde{p}\left(x\right)\log\frac{1}{\tilde{p}\left(x\right)}+\tilde{p}\left(x\right)\right)d\mu \left(x\right)-1.$$

**Remark** **4.** *In general, we can consider two unnormalized positive densities $\tilde{p}\left(x\right)$ and $\tilde{q}\left(x\right)$. Let $p\left(x\right)=\frac{\tilde{p}\left(x\right)}{Z_{p}}$ and $q\left(x\right)=\frac{\tilde{q}\left(x\right)}{Z_{q}}$ denote their corresponding normalized densities (with normalizing factors $Z_{p}=\int \tilde{p}\;d\mu$ and $Z_{q}=\int \tilde{q}\;d\mu$); then, the KLD between $\tilde{p}$ and $\tilde{q}$ can be expressed using the KLD between their normalized densities and normalizing factors, as follows:*

(17) $$D_{\mathrm{KL}}\left(\tilde{p}:\tilde{q}\right)=Z_{p}\;\left(D_{\mathrm{KL}}\left(p:q\right)+\log\frac{Z_{p}}{Z_{q}}\right)+Z_{q}-Z_{p}.\;$$

*Similarly, we have*

(18) $$\begin{array}{ccc} H^{\times }\left(\tilde{p}:\tilde{q}\right) & = & Z_{p}\;H^{\times }\left(p:q\right)-Z_{p}\log Z_{q}+Z_{q}-1, \end{array}$$

(19) $$\begin{array}{ccc} H(\tilde{p}) & = & Z_{p}\;H\left(p\right)-Z_{p}\log Z_{p}+Z_{p}-1, \end{array}$$

*and $D_{\mathrm{KL}}\left(\tilde{p}:\tilde{q}\right)=H^{\times }\left(\tilde{p}:\tilde{q}\right)-H\left(\tilde{p}\right)$.*

Notice that Equation (17) allows us to derive the following identity between $B_{Z}$ and $B_{F}$:

(20) $$\begin{array}{ccc} B_{Z}\left(\theta_{2}:\theta_{1}\right) & = & Z\left(\theta_{1}\right)\;B_{F}\left(\theta_{2}:\theta_{1}\right)+Z\left(\theta_{1}\right)\log\frac{Z(\theta_{1})}{Z(\theta_{2})}+Z\left(\theta_{2}\right)-Z\left(\theta_{1}\right), \end{array}$$

(21) $$\begin{array}{ccc} & = & \exp\left(F\left(\theta_{1}\right)\right)\;B_{F}\left(\theta_{2}:\theta_{1}\right)+\left(\exp F\left(\theta_{1}\right)\right)\left(F\left(\theta_{1}\right)-F\left(\theta_{2}\right)\right)+\exp\left(F\left(\theta_{2}\right)\right)-\exp\left(F\left(\theta_{1}\right)\right). \end{array}$$

Let $D_{\mathrm{skl}}\left(a:b\right)=a\log\frac{a}{b}+b-a$ be the scalar KLD for a>0 and b>0. Then, we can rewrite Equation (17) as

$$D_{\mathrm{KL}}\left(\tilde{p}:\tilde{q}\right)=Z_{p}\;D_{\mathrm{KL}}\left(p:q\right)+D_{\mathrm{skl}}\left(Z_{p}:Z_{q}\right),$$

and we have

$$B_{Z}\left(\theta_{2}:\theta_{1}\right)=Z\left(\theta_{1}\right)\;B_{F}\left(\theta_{2}:\theta_{1}\right)+D_{\mathrm{skl}}\left(Z\left(\theta_{1}\right):Z\left(\theta_{2}\right)\right).$$

In addition, the KLD between the unnormalized densities $\tilde{p}$ and $\tilde{q}$ with support X can be written as a definite integral of a scalar Bregman divergence:

$$D_{\mathrm{KL}}\left(\tilde{p}:\tilde{q}\right)=\int_{X}D_{\mathrm{skl}}\left(\tilde{p}\left(x\right):\tilde{q}\left(x\right)\right)\;d\mu \left(x\right)=\int_{X}B_{f_{\mathrm{skl}}}\left(\tilde{p}\left(x\right):\tilde{q}\left(x\right)\right)\;d\mu \left(x\right),\;$$

where $f_{\mathrm{skl}}\left(x\right)=x\log x-x$. Because $B_{f_{\mathrm{skl}}}\left(a:b\right)\geq 0\forall a>0,b>0$, we can deduce that $D_{\mathrm{KL}}\left(\tilde{p}:\tilde{q}\right)\geq 0$ with equality iff $\tilde{p}\left(x\right)=\tilde{q}\left(x\right)$μ almost everywhere.

Notice that $B_{Z}\left(\theta_{2}:\theta_{1}\right)=Z\left(\theta_{1}\right)\;B_{F}\left(\theta_{2}:\theta_{1}\right)+D_{\mathrm{skl}}\left(Z\left(\theta_{1}\right):Z\left(\theta_{2}\right)\right)$ can be interpreted as the sum of two divergences, that is, a conformal Bregman divergence with a scalar Bregman divergence.

**Remark** **5.** *Consider the KLD between the normalized $p_{\theta_{1}}$ and unnormalized ${\tilde{p}}_{\theta_{2}}$ densities of the same exponential family. In this case, we have*

(22) $$\begin{array}{ccc} D_{\mathrm{KL}}\left(p_{\theta_{1}}:{\tilde{p}}_{\theta_{2}}\right) & = & B_{F}\left(\theta_{2}:\theta_{1}\right)-\log Z\left(\theta_{2}\right)+Z\left(\theta_{2}\right)-1,\\ & = & Z\left(\theta_{2}\right)-1-F\left(\theta_{1}\right)-\langle \theta_{2}-\theta_{1},\nabla F\left(\theta_{2}\right)\rangle , \end{array}$$

(23) $$\begin{array}{ccc} & = & B_{Z-1,F}\left(\theta_{2}:\theta_{1}\right). \end{array}$$

*The divergence $B_{Z-1,F}$ is a dual Bregman pseudo-divergence* [28]*:*

$$B_{F_{1},F_{2}}\left(\theta_{1}:\theta_{2}\right)=F_{1}\left(\theta_{1}\right)-F_{2}\left(\theta_{2}\right)-\langle \theta_{1}-\theta_{2},\nabla F_{2}\left(\theta_{2}\right)\rangle ,$$

*for $F_{1}$ and $F_{2}$ that are two strictly convex and smooth functions such that $F_{1}\geq F_{2}$. Indeed, we can check that generators $F_{1}\left(\theta \right)=Z\left(\theta \right)-1$ and $F_{2}\left(\theta \right)=F\left(\theta \right)$ are both Bregman generators; then, we have $F_{1}\left(\theta \right)\geq F_{2}\left(\theta \right)$, as $e^{x}\geq x+1$ for all x (with equality when x=0), i.e., Z(θ)−1≥F(θ).*

*More generally, the α-divergences between $p_{\theta_{1}}$ and ${\tilde{p}}_{\theta_{2}}$ can be written as*

(24) $$D_{\alpha }\left(p_{\theta_{1}}:{\tilde{p}}_{\theta_{2}}\right)=\frac{1}{\alpha (1-\alpha )}\left(\alpha Z\left(\theta_{1}\right)+\left(1-\alpha \right)-\frac{Z(\alpha \theta_{1}+\left(1-\alpha \right)\theta_{2})}{Z(\theta_{2})}\right),$$

*with the (signed) α-skewed Bhattacharyya distances provided by*

$$D_{B,\alpha }\left(p_{\theta_{1}}:{\tilde{p}}_{\theta_{2}}\right)=\log Z\left(\theta_{2}\right)-\log Z\left(\alpha \theta_{1}+\left(1-\alpha \right)\theta_{2}\right).$$

Let us illustrate Proposition 5 with some examples.

**Example** **1.** *Consider the family of exponential distributions $E=\{p_{\lambda }\left(x\right)=1_{x\geq 0}\;\lambda \exp\left(-\lambda x\right)\}$, where E is an exponential family with a natural parameter θ=λ, parameter space Θ=R>0, sufficient statistic t(x)=−x. The partition function is $Z\left(\theta \right)=\frac{1}{\theta }$, with $Z^{\prime }\left(\theta \right)=-\frac{1}{\theta^{2}}$ and $Z^{\prime \prime }\left(\theta \right)=\frac{2}{\lambda^{3}}>0$, while the cumulant function is F(θ)=logZ(θ)=−logθ with moment parameter $\eta =E_{p_{\lambda }}\left[t\left(x\right)\right]=F^{\prime }\left(\theta \right)=-\frac{1}{\theta }$. The α-divergences between two unnormalized exponential distributions are*

(25) $$D_{\alpha }\left({\tilde{p}}_{\lambda_{1}}:{\tilde{p}}_{\lambda_{2}}\right)=\left\{\begin{array}{cc} \frac{1}{\alpha (1-\alpha )}J_{Z,\alpha }\left(\theta_{1}:\theta_{2}\right)=\frac{{\left(\lambda_{1}-\lambda_{2}\right)}^{2})}{\alpha \lambda_{1}^{2}\lambda_{2}+\left(1-\alpha \right)\lambda_{1}\lambda_{2}^{2}} & \alpha \notin \{0,1\}\\ D_{\mathrm{KL}}\left({\tilde{p}}_{\lambda_{2}}:{\tilde{p}}_{\lambda_{1}}\right)=B_{Z}\left(\theta_{1}:\theta_{2}\right)=\frac{{\left(\lambda_{1}-\lambda_{2}\right)}^{2}}{\lambda_{1}\lambda_{2}^{2}} & \alpha =0,\\ 4\;J_{Z}\left(\theta_{1},\theta_{2}\right)=\frac{{\left(\lambda_{1}-\lambda_{2}\right)}^{2}}{2(\lambda_{1}\lambda_{2}^{2}+\lambda_{1}^{2}\lambda_{2})} & \alpha =\frac{1}{2},\\ D_{\mathrm{KL}}\left({\tilde{p}}_{\lambda_{1}}:{\tilde{p}}_{\lambda_{2}}\right)=B_{Z}\left(\theta_{2}:\theta_{1}\right)=\frac{{\left(\lambda_{1}-\lambda_{2}\right)}^{2}}{\lambda_{2}\lambda_{1}^{2}} & \alpha =1 \end{array}.\right.$$

**Example** **2.** *Consider the family of univariate centered normal distributions with ${\tilde{p}}_{\sigma^{2}}\left(x\right)\propto \exp\left(-\frac{x^{2}}{2\sigma^{2}}\right)$ and partition function $Z\left(\sigma^{2}\right)=\sqrt{2\pi \sigma^{2}}$ such that $p_{\sigma^{2}}\left(x\right)=\frac{1}{Z(\sigma^{2})}\;{\tilde{p}}_{\sigma^{2}}\left(x\right)=\frac{1}{\sqrt{2\pi \sigma^{2}}}\exp\left(-\frac{x^{2}}{2\sigma^{2}}\right)$. Here, we have a natural parameter $\theta =\frac{1}{\sigma^{2}}\in \Theta =R_{>0}$ and sufficient statistic $t\left(x\right)=-\frac{x^{2}}{2}$. The partition function expressed with the natural parameter is $Z\left(\theta \right)=\sqrt{\frac{2\pi }{\theta }}$, with $Z^{\prime }\left(\theta \right)=-\sqrt{\frac{\pi }{2}}\theta^{-\frac{3}{2}}$ and $Z^{\prime \prime }\left(\theta \right)=\frac{3\sqrt{\pi }}{2^{\frac{3}{2}}}\theta^{-\frac{5}{2}}>0$ (strictly convex on *Θ*). The unnormalized KLD between ${\tilde{p}}_{\sigma_{1}^{2}}$ and ${\tilde{p}}_{\sigma_{2}^{2}}$ is*

$$D_{\mathrm{KL}}\left({\tilde{p}}_{\sigma_{1}^{2}}:{\tilde{p}}_{\sigma_{2}^{2}}\right)=B_{Z}\left(\theta_{2}:\theta_{1}\right)=\sqrt{\frac{\pi }{2}}\;\left(2\sigma_{2}-3\sigma_{1}+\frac{\sigma_{1}^{3}}{\sigma_{2}^{2}}\right).\;$$

*We can check that we have $D_{\mathrm{KL}}\left({\tilde{p}}_{\sigma^{2}}:{\tilde{p}}_{\sigma^{2}}\right)=0$.*

*For the Hellinger divergence, we have*

$$D_{H}^{2}\left({\tilde{p}}_{\sigma_{1}^{2}}:{\tilde{p}}_{\sigma_{2}^{2}}\right)=J_{Z}\left(\theta_{1},\theta_{2}\right)=\sqrt{\frac{\pi }{2}}\left(\sigma_{1}+\sigma_{2}\right)-2\sqrt{\pi }\frac{\sigma_{1}\sigma_{2}}{\sqrt{\sigma_{1}^{2}+\sigma_{2}^{2}}},\;$$

*and we can check that $D_{H}\left({\tilde{p}}_{\sigma^{2}}:{\tilde{p}}_{\sigma^{2}}\right)=0$.*

*Consider the family of the d-variate case of centered normal distributions with unnormalized density*

$${\tilde{p}}_{\Sigma }\left(x\right)\propto \exp\left(-\frac{1}{2}x^{⊤}\Sigma^{-1}x\right)=\exp\left(-\frac{1}{2}\mathrm{tr}\left(x^{⊤}\Sigma^{-1}x\right)\right)=\exp\left(-\frac{1}{2}\mathrm{tr}\left(xx^{⊤}\Sigma^{-1}\right)\right)$$

*obtained using the matrix trace cyclic property, where *Σ* is the covariance matrix. Here, we have $\theta =\Sigma^{-1}$ (precision matrix) and $\Theta ={\mathrm{Sym}}_{++}\left(d\right)$ for $t\left(x\right)=-\frac{1}{2}xx^{⊤}$, with the matrix inner product $\langle A,B\rangle =\mathrm{tr}\left(A^{⊤}B\right)$. The partition function $Z\left(\Sigma \right)={\left(2\pi \right)}^{\frac{d}{2}}\sqrt{\det(\Sigma )}$ expressed with the natural parameter is $Z\left(\theta \right)={\left(2\pi \right)}^{\frac{d}{2}}\sqrt{\frac{1}{\det(\theta )}}$. This is a convex function with*

$$\nabla Z\left(\theta \right)=-\frac{1}{2}{\left(2\pi \right)}^{\frac{d}{2}}\frac{\nabla_{\theta }\det\left(\theta \right)}{\det{\left(\theta \right)}^{\frac{3}{2}}}=-\frac{1}{2}{\left(2\pi \right)}^{\frac{d}{2}}\frac{\theta^{-1}}{\det{\left(\theta \right)}^{\frac{1}{2}}},\;$$

*as $\nabla_{\theta }\det\left(\theta \right)=\det\left(\theta \right)\theta^{-⊤}$ using matrix calculus.*

*Now, consider the family of univariate normal distributions*

$$E=\left\{p_{\mu ,\sigma^{2}}\left(x\right)=\frac{1}{\sqrt{2\pi \sigma^{2}}}\exp\left(-\frac{1}{2}{\left(\frac{x-\mu }{\sigma }\right)}^{2}\right)\right\}.$$

*Let $\theta =\left(\theta_{1}=\frac{1}{\sigma_{2}},\theta_{2}=\frac{\mu }{\sigma^{2}}\right)$ and*

$$Z\left(\theta_{1},\theta_{2}\right)=\sqrt{\frac{2\pi }{\theta_{1}}}\;\exp\left(\frac{1}{2}\frac{\theta_{2}^{2}}{\theta_{1}}\right).\;$$

*The unnormalized densities are ${\tilde{p}}_{\theta }\left(x\right)=\exp\left(-\frac{\theta_{1}x^{2}}{2}+x\theta_{2}\right)$, and we have*

$$\nabla Z\left(\theta \right)=\left[\begin{array}{c} \sqrt{\frac{\pi }{2}}\frac{\left(\theta_{1}+\theta_{2}^{2}\right)\exp\left(\frac{\theta_{2}^{2}}{2\theta_{1}}\right)}{\theta_{1}^{\frac{5}{2}}}\\ \sqrt{2\pi }\frac{\theta_{2}\exp\left(\frac{\theta_{2}^{2}}{2\theta_{1}}\right)}{\theta_{1}^{\frac{3}{2}}} \end{array}\right].$$

*It follows that $D_{\mathrm{KL}}\left[{\tilde{p}}_{\theta }:{\tilde{p}}_{\theta^{\prime }}\right]=B_{Z}\left(\theta^{\prime }:\theta \right)$.*

## 5. Deforming Convex Functions and Their Induced Dually Flat Spaces

### 5.1. Comparative Convexity

The log-convexity can be interpreted as a special case of comparative convexity with respect to a pair (M,N) of comparable weighted means [9], as follows.

A function *Z* is (M,N)-convex if and only if for α∈[0,1] we have

(26) Z(M(x,y;α,1−α))≤N(Z(x),Z(y);α,1−α),

and is strictly (M,N)-convex iff we have strict inequality for α∈(0,1) and x≠y. Furthermore, a function *Z* is (strictly) (M,N)-concave if −Z is (strictly) (M,N)-convex.

Log-convexity corresponds to (A,G)-convexity, i.e., convexity with respect to the weighted arithmetical and geometrical means defined respectively by A(x,y;α,1−α)=αx+(1−α)y and $G\left(x,y;\alpha ,1-\alpha \right)=x^{\alpha }y^{1-\alpha }$. Ordinary convexity is (A,A)-convexity.

A weighted quasi-arithmetical mean [34] (also called a Kolmogorov–Nagumo mean [35]) is defined for a continuous and strictly increasing function *h* by

$$M_{h}\left(x,y;\alpha ,1-\alpha \right)=h^{-1}\left(\alpha h\left(x\right)+\left(1-\alpha \right)h\left(x\right)\right).\;$$

We let $M_{h}\left(x,y\right)=M_{h}\left(x,y;\frac{1}{2},\frac{1}{2}\right)$. Quasi-arithmetical means include the arithmetical mean obtained for h(u)=id(u)=u and the geometrical mean for h(u)=log(u), and more generally power means

$$M_{p}\left(x,y;\alpha ,1-\alpha \right)={\left(\alpha x^{p}+\left(1-\alpha \right)y^{p}\right)}^{\frac{1}{p}}=M_{h_{p}}\left(x,y;\alpha ,1-\alpha \right),\;p\neq 0,\;$$

which are quasi-arithmetical means obtained for the family of generators $h_{p}\left(u\right)=\frac{u^{p}-1}{p}$ with inverse $h_{p}^{-1}\left(u\right)={\left(1+up\right)}^{\frac{1}{p}}$. In the limit p→0, we have $M_{0}\left(x,y\right)=G\left(x,y\right)$ for the generator $\lim_{p\to 0}h_{p}\left(u\right)=h_{0}\left(u\right)=\log u$.

**Proposition** **6** ([36,37]). *A function Z(θ) is strictly $\left(M_{\rho },M_{\tau }\right)$-convex with respect to two strictly increasing smooth functions ρ and τ if and only if the function $F=\tau \circ Z\circ \rho^{-1}$ is strictly convex.*

Notice that the set of strictly increasing smooth functions form a non-Abelian group, with the group operation as the function composition, the neutral element as the identity function, and the inverse element as the functional inverse function.

Because log-convexity is $\left(A=M_{\mathrm{id}},G=M_{\log}\right)$-convexity, a function *Z* is strictly log-convex iff $\log\circ Z\circ {\mathrm{id}}^{-1}=\log\circ Z$ is strictly convex. We have

$$Z=\tau^{-1}\circ F\circ \rho \Leftrightarrow F=\tau \circ Z\circ \rho^{-1}.\;$$

Starting from a given convex function F(θ), we can deform the function F(θ) to obtain a function Z(θ) using two strictly monotone functions τ and ρ: $Z\left(\theta \right)=\tau^{-1}\left(F\left(\rho \left(\theta \right)\right)\right)$.

For a $\left(M_{\rho },M_{\tau }\right)$-convex function Z(θ) which is also strictly convex, we can define a pair of Bregman divergences $B_{Z}$ and $B_{F}$ with $F\left(\theta \right)=\tau (Z\left(\rho^{-1}\left(\theta \right)\right))$ and a corresponding pair of skewed Jensen divergences.

Thus, we have the following generic deformation scheme.

$$\underset{(M_{\rho^{-1}},M_{\tau^{-1}})\text{-}convex\;when\;Z\;is\;convex}{\underset{︸}{F=\tau \circ Z\circ \rho^{-1}}}\;\;\underset{\left(\rho^{-1},\tau^{-1}\right)\text{-deformation}}{\underset{⇌}{\left(\rho ,\tau \right)\text{-deformation}}}\;\;\underset{(M_{\rho },M_{\tau })\text{-}convex\;when\;F\;is\;convex}{\underset{︸}{Z=\tau^{-1}\circ F\circ \rho }}$$

In particular, when the function *Z* is deformed by strictly increasing the power functions $h_{p_{1}}$ and $h_{p_{2}}$ for $p_{1}$ and $p_{2}$ in R as

$$Z_{p_{1},p_{2}}=h_{p_{2}}\circ Z\circ h_{p_{1}}^{-1},$$

then $Z_{p_{1},p_{2}}$ is strictly convex when it is strictly $\left(M_{p_{1}},M_{p_{2}}\right)$-convex, and as such induces corresponding Bregman and Jensen divergences.

**Example** **3.** *Consider the partition function $Z\left(\theta \right)=\frac{1}{\theta }$ of the exponential distribution family (θ>0 with $\Theta =R_{>0}$). Let $Z_{p}\left(\theta \right)=\left(h_{p}\circ Z\right)\left(\theta \right)=\frac{\theta^{-p}-1}{p}$; then, we have $Z_{p}^{\prime \prime }\left(\theta \right)=\left(1+p\right)\frac{1}{\theta^{2+p}}>0$ when p>−1. Thus, we can deform Z smoothly by $Z_{p}$ while preserving the convexity by ranging p from −1 to +∞. In this way, we obtain a corresponding family of Bregman and Jensen divergences.*

The proposed convex deformation using quasi-arithmetical mean generators differs from the interpolation of convex functions using the technique of proximal averaging [38].

Note that in [37] the comparative convexity with respect to a pair of quasi-arithmetical means $\left(M_{\rho },M_{\tau }\right)$ is used to define a $\left(M_{\rho },M_{\tau }\right)$-Bregman divergence, which turns out to be equivalent to a conformal Bregman divergence on the ρ-embedding of the parameters.

### 5.2. Dually Flat Spaces

We start with a refinement of the class of convex functions used to generate dually flat spaces.

**Definition** **2** (Legendre type function [39]). *(Θ,F) is of Legendre type if the function F:Θ→R is strictly convex and differentiable with Θ≠∅ and*

(27) $$\lim_{\lambda \to 0}\frac{d}{d\lambda }F\left(\lambda \theta +\left(1-\lambda \right)\bar{\theta }\right)=-\infty ,\;\forall \theta \in \Theta ,\forall \bar{\theta }\in \partial \Theta .$$

Legendre-type functions F(Θ) admit a convex conjugate $F^{*}\left(\eta \right)$ via the Legendre transform $F^{*}\left(\eta \right)=\sup_{\theta \in \Theta }\langle \theta ,\eta \rangle -F\left(\theta \right)$:

$$F^{*}\left(\eta \right)=\langle \nabla F^{-1}\left(\eta \right),\eta \rangle -F\left(\nabla F^{-1}\left(\eta \right)\right).\;$$

**Figure 2:**
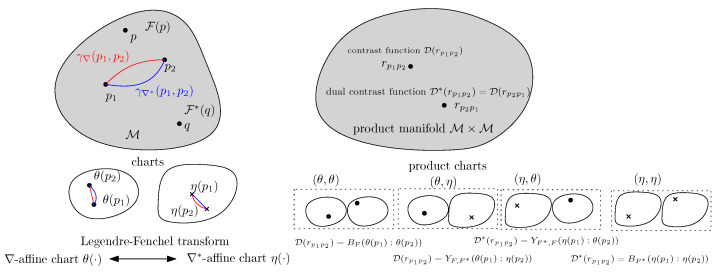
The canonical divergence D and dual canonical divergence $D^{*}$ on a dually flat space M equipped with potential functions F and $F^{*}$ can be viewed as single-parameter contrast functions on the product manifold M×M: The divergence D can be expressed using either the θ×θ-coordinate system as a Bregman divergence or the mixed θ×η-coordinate system as a Fenchel–Young divergence. Similarly, the dual divergence D can be expressed using either the η×η-coordinate system as a dual Bregman divergence or the mixed η×θ-coordinate system as a dual Fenchel–Young divergence.

A smooth and strictly convex function (Θ,F(θ)) of Legendre type induces a dually flat space [1] M, i.e., a smooth Hessian manifold [40] with a single global chart (Θ,θ(·)) [1]. A canonical divergence D(p:q) between two points *p* and *q* of M is viewed as a single-parameter contrast function [41] $D(r_{pq})$ on the product manifold M×M. The canonical divergence and its dual canonical divergence $D^{*}\left(r_{qp}\right)=D\left(r_{pq}\right)$ can be expressed equivalently as either dual Bregman divergences or dual Fenchel–Young divergences (Figure 2):

$$\begin{array}{ccc} D(r_{pq}) & = & B_{F}\left(\theta \left(p\right):\theta \left(q\right)\right)=Y_{F,F^{*}}\left(\theta \left(p\right):\eta \left(q\right)\right),\\ & = & D^{*}\left(r_{qp}\right)=B_{F^{*}}\left(\eta \left(q\right):\eta \left(p\right)\right)=Y_{F^{*},F}\left(\eta \left(q\right):\theta \left(p\right)\right), \end{array}$$

where $Y_{F,F^{*}}$ is the Fenchel–Young divergence:

$$Y_{F,F^{*}}\left(\theta \left(p\right):\eta \left(q\right)\right)=F\left(\theta \left(p\right)\right)+F^{*}\left(\eta \left(q\right)\right)-\langle \theta (p),\eta (q)\rangle .$$

We have the dual global coordinate system η=∇F(θ) and the domain H={∇F(θ):θ∈Θ} which defines the dual Legendre-type potential function $\left(H,F^{*}\left(\eta \right)\right)$. The Legendre-type function ensures that ${F^{*}}^{*}=F$ (a sufficient condition is to have *F* be convex and lower semi-continuous [42]).

A manifold M is called dually flat, as the torsion-free affine connections ∇ and $\nabla^{*}$ induced by the potential functions F(θ) and $F^{*}\left(\eta \right)$ linked with the Legendre–Fenchel transformation are flat [1], that is, their Christoffel symbols vanishes in the dual coordinate system: Γ(θ)=0 and $\Gamma^{*}\left(\eta \right)=0$.

The Legendre-type function (Θ,F(θ)) is not defined uniquely; the function $\bar{F}\left(\bar{\theta }\right)=F\left(A\theta +b\right)+C\theta +d$ with $\bar{\theta }=A\theta +b$ for *A* and *C* invertible matrices and *b* and *d* vectors defines the same dually flat space with the same canonical divergence D(p,q):

$$D\left(p:q\right)=B_{F}\left(\theta \left(p\right):\theta \left(q\right)\right)=B_{\bar{F}}\left(\bar{\theta }\left(p\right):\bar{\theta }\left(q\right)\right).\;$$

Thus, a log-convex Legendre-type function Z(θ) induces two dually flat spaces by considering the DFSs induced by Z(θ) and F(θ)=logZ(θ). Let the gradient maps be η=∇Z(θ) and $\tilde{\eta }=\nabla F\left(\theta \right)=\frac{\eta }{Z(\theta )}$.

When F(θ) is chosen as the cumulant function of an exponential family, the Bregman divergence $B_{F}\left(\theta_{1}:\theta_{2}\right)$ can be interpreted as a statistical divergence between corresponding probability densities, meaning that the Bregman divergence amounts to the reverse Kullback–Leibler divergence: $B_{F}\left(\theta_{1}:\theta_{2}\right)=D_{\mathrm{KL}}^{*}\left(p_{\theta_{1}}:p_{\theta_{2}}\right)$, where $D_{\mathrm{KL}}^{*}$ is the reverse KLD.

Notice that deforming a convex function F(θ) into F(ρ(θ)) such that F∘ρ remains strictly convex has been considered by Yoshizawa and Tanabe [43] to build a two-parameter deformation $\rho_{\alpha ,\beta }$ of the dually flat space induced by the cumulant function F(θ) of the multivariate normal family. Additionally, see the method of Hougaard [44] for obtaining other exponential families from a given exponential family.

Thus, in general, there are many more dually flat spaces with corresponding divergences and statistical divergences than the usually considered exponential family manifold [5] induced by the cumulant function. It is interesting to consider their use in information sciences.

## 6. Conclusions and Discussion

For machine learning practioners, it is well known that the Kullback–Leibler divergence (KLD) between two probability densities $p_{\theta_{1}}$ and $p_{\theta_{2}}$ of an exponential family with cumulant function *F* (free energy in thermodynamics) amounts to a reverse Bregman divergence [26] induced by *F*, or equivalently to a reverse Fenchel–Young divergence [27]

$$D_{\mathrm{KL}}\left(p_{\theta_{1}}:p_{\theta_{2}}\right)=B_{F}\left(\theta_{2}:\theta_{1}\right)=Y_{F,F^{*}}\left(\theta_{2}:\eta_{1}\right),\;$$

where η=∇F(θ) is the dual moment or expectation parameter.

In this paper, we have shown that the KLD as extended to positive unnormalized densities ${\tilde{p}}_{\theta_{1}}$ and ${\tilde{p}}_{\theta_{2}}$ of an exponential family with a convex partition function Z(θ) (Laplace transform) amounts to a reverse Bregman divergence induced by *Z*, or equivalently to a reverse Fenchel–Young divergence

$$D_{\mathrm{KL}}\left({\tilde{p}}_{\theta_{1}}:{\tilde{p}}_{\theta_{2}}\right)=B_{Z}\left(\theta_{2}:\theta_{1}\right)=Y_{Z,Z^{*}}\left(\theta_{2}:{\tilde{\eta }}_{1}\right),\;$$

where $\tilde{\eta }=\nabla Z\left(\theta \right)$.

More generally, we have shown that the scaled α-skewed Jensen divergences induced by the cumulant and partition functions between natural parameters coincide with the scaled α-skewed Bhattacharyya distances between probability densities and the α-divergences between unnormalized densities, respectively:

$$\begin{array}{ccc} D_{B,\alpha }^{s}\left(p_{\theta_{1}}:p_{\theta_{2}}\right) & = & J_{F,\alpha }^{s}\left(\theta_{1}:\theta_{2}\right),\\ D_{\alpha }\left({\tilde{p}}_{\theta_{1}}:{\tilde{p}}_{\theta_{2}}\right) & = & J_{Z,\alpha }^{s}\left(\theta_{1}:\theta_{2}\right).\; \end{array}$$

We have noted that the partition functions *Z* of exponential families are both convex and log-convex, and that the corresponding cumulant functions are both convex and exponentially convex.

**Figure 3:**
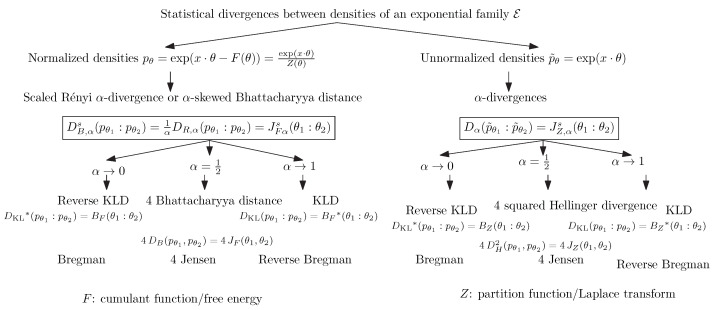
Statistical divergences between normalized $p_{\theta }$ and unnormalized ${\tilde{p}}_{\theta }$ densities of an exponential family E with corresponding divergences between their natural parameters. Without loss of generality, we consider a natural exponential family (i.e., t(x)=x and k(x)=0) with cumulant function *F* and partition function *Z*, with $J_{F}$ and $B_{F}$ respectively denoting the Jensen and Bregman divergences induced by the generator *F*. The statistical divergences $D_{R,\alpha }$ and $D_{B,\alpha }$ denote the Rényi α-divergences and skewed α-Bhattacharyya distances, respectively. The superscript “s” indicates rescaling by the multiplicative factor $\frac{1}{\alpha (1-\alpha )}$, while the superscript “*” denotes the reverse divergence obtained by swapping the parameter order.

Figure 3 summarizes the relationships between statistical divergences and between the normalized and unnormalized densities of an exponential family, as well as the corresponding divergences between their natural parameters. Notice that Brekelmans and Nielsen [45] considered deformed uni-order likelihood ratio exponential families (LREFs) for annealing paths and obtained an identity for the α-divergences between unnormalized densities and Bregman divergences induced by multiplicatively scaled partition functions.

Because the log-convex partition function is also convex, we have generalized the principle of building pairs of convex generators using the comparative convexity with respect to a pair of quasi-arithmetical means, and have further discussed the induced dually flat spaces and divergences. In particular, by considering the convexity-preserving deformations obtained by power mean generators, we have shown how to obtain a family of convex generators and dually flat spaces. Notice that some parametric families of Bregman divergences, such as the α-divergences [46], β-divergences [47], and *V*-geometry [48] of symmetric positive-definite matrices, yield families of dually flat spaces.

Banerjee et al. [49] proved a duality between regular exponential families and a subclass of Bregman divergences, which they accordingly termed regular Bregman divergences. In particular, this duality allows the Maximum Likelihood Estimator (MLE) of an exponential family with a cumulant function *F* to be viewed as a right-sided Bregman centroid with respect to the Legendre–Fenchel dual $F^{*}$. In [50], the scope of this duality was further extended for arbitrary Bregman divergences by introducing a class of generalized exponential families.

Concave deformations have been recently studied in [51], where the authors introduced the $\log_{\phi }$-concavity induced by a positive continuous function ϕ generating a deformed logarithm $\log_{\phi }$ as the $\left(A,\log_{\phi }\right)$-comparative concavity (Definition 1.2 in [51]), as well as the weaker notion of *F*-concavity which corresponds to the (A,F)-concavity (Definition 2.1 in [51], requiring strictly increasing functions *F*). Our deformation framework $Z=\tau^{-1}\circ F\circ \rho$ is more general, as it is double-sided. We jointly deform the function *F* by $F_{\tau }=\tau^{-1}\circ F$ and its argument θ by $\theta_{\rho }=\rho \left(\theta \right)$.

Exponentially concave functions have been considered as generators of *L*-divergences in [24]; α-exponentially concave functions *G* such that exp(αG) are concave for α>0 generalize the *L*-divergences to $L_{\alpha }$-divergences, which can be expressed equivalently using a generalization of the Fenchel–Young divergence based on the *c*-transforms [24]. When α<0, exponentially convex functions are considered instead of exponentially concave functions. The information geometry induced by $L_{\alpha }$-divergences are dually projectively flat with constant curvature, and reciprocally possess a dually projectively flat structure with constant curvature, inducing (locally) a canonical $L_{-\alpha }$-divergence. Wong and Zhang [52] investigated a one-parameter deformation of convex duality, called λ-duality, by considering functions *f* such that $\frac{1}{\lambda }\left(e^{\lambda f}-1\right)$ are convex for λ≠0. They defined the λ-conjugate transform as a particular case of the *c*-transform [24] and studie the information geometry of the induced λ-logarithmic divergences. The λ-duality yields a generalization of exponential and mixture families to λ-exponential and λ-mixture families related to the Rényi divergence.

Finally, certain statistical divergences, called projective divergences, are invariant under rescaling, and as such can define dissimilarities between non-normalized densities. For example, the γ-divergences [32] $D_{\gamma }$ are such that $D_{\gamma }\left(p:q\right)=D_{\gamma }\left(\tilde{p}:\tilde{q}\right)$ (with γ-divergences tending to the KLD when γ→0) or the Cauchy–Schwarz divergence [53].

## Data Availability

No new data were created or analyzed in this study. Data sharing is not applicable to this article.
